# Research on the Optimum Water Content of Detecting Soil Nitrogen Using Near Infrared Sensor

**DOI:** 10.3390/s17092045

**Published:** 2017-09-07

**Authors:** Yong He, Shupei Xiao, Pengcheng Nie, Tao Dong, Fangfang Qu, Lei Lin

**Affiliations:** 1 College of Biosystems Engineering and Food Science, Zhejiang University, Hangzhou 310058, China; yhe@zju.edu.cn (Y.H.); xsp941230@163.com (S.X.); dt2016@zju.edu.cn (T.D.); ffqu@zju.edu.cn (F.Q.); linlei2016@zju.edu.cn (L.L.); 2 Key Laboratory of Spectroscopy Sensing, Ministry of Agriculture, Zhejiang University, Hangzhou 310058, China; 3 State Key Laboratory of Modern Optical Instrumentation, Zhejiang University, Hangzhou 310058, China

**Keywords:** nitrogen, near infrared sensor, water content, drying time, PLS, UVE

## Abstract

Nitrogen is one of the important indexes to evaluate the physiological and biochemical properties of soil. The level of soil nitrogen content influences the nutrient levels of crops directly. The near infrared sensor can be used to detect the soil nitrogen content rapidly, nondestructively, and conveniently. In order to investigate the effect of the different soil water content on soil nitrogen detection by near infrared sensor, the soil samples were dealt with different drying times and the corresponding water content was measured. The drying time was set from 1 h to 8 h, and every 1 h 90 samples (each nitrogen concentration of 10 samples) were detected. The spectral information of samples was obtained by near infrared sensor, meanwhile, the soil water content was calculated every 1 h. The prediction model of soil nitrogen content was established by two linear modeling methods, including partial least squares (PLS) and uninformative variable elimination (UVE). The experiment shows that the soil has the highest detection accuracy when the drying time is 3 h and the corresponding soil water content is 1.03%. The correlation coefficients of the calibration set are 0.9721 and 0.9656, and the correlation coefficients of the prediction set are 0.9712 and 0.9682, respectively. The prediction accuracy of both models is high, while the prediction effect of PLS model is better and more stable. The results indicate that the soil water content at 1.03% has the minimum influence on the detection of soil nitrogen content using a near infrared sensor while the detection accuracy is the highest and the time cost is the lowest, which is of great significance to develop a portable apparatus detecting nitrogen in the field accurately and rapidly.

## 1. Introduction

Soil is the substrate supplying the nutrient sources for most crops, which is also a primary material and an energy exchange site. Soil nitrogen content is the indicator to evaluate soil fertility, the level of nitrogen influences the crops’ growth and development directly. Therefore, obtaining the soil nutrient information rapidly and accurately, such as nitrogen content, is of great importance for optimizing the amount of fertilizer in real time [1,2]. The traditional chemical method of detecting soil nitrogen content, such as Dumas combustion, achieves a high accuracy [3]. Nevertheless, the whole detection process is complex, destructive, and time-consuming with a low economic benefit. Detecting the soil nitrogen content by near infrared sensor is widely used, which meets the demands of precision agriculture for involving rapid, accurate, and time-saving methods for quick control and prediction of soil quality [4].

In recent years, researchers have applied near infrared sensor to detect soil nitrogen content with some achievements. He et al. used near infrared sensor to detect soil nitrogen, phosphorus, potassium, organic matter, and PH value, indicating that the reflectance spectrum of soil had a close correlation with soil total nitrogen content, which could be applied to predict soil nitrogen and organic matter [5,6,7]. Nie et al. detected a soil nitrogen content prepared with three different pretreatment methods by near infrared sensor. It was proved that the soil with the strictest pretreatment (dried, ground, sieved, and pressed) had the highest accuracy in predicting the soil nitrogen content [8]. Antonios Morellos et al. improved the algorithm accuracy by means of two multivariate methods including principal component regression (PCR) and partial least squares regression (PLSR), and two machine learning methods including least squares support vector machines (LS-SVM) and Cubist, revealing that the Cubist method provided the best [9]. Liu compared the performance of four spectral transformation strategies in the removal of soil moisture effects on total nitrogen estimation. The results demonstrated that the value of the orthogonal signal correction (OSC) and generalized least squares weighting (GLSW) in eliminating the effects of moisture on the total nitrogen estimation [10]. Zhang et al. explored six sensitive wavebands using wavelet decomposition and continuous removal method, both of which performed well in reducing the interference of soil moisture and predicting the soil total nitrogen content in real time [11].

The prediction accuracy of soil nitrogen content can be improved by different methods, namely, soil pretreatment, spectral reflectance data processing, feature band selection, and algorithm optimization [12,13,14]. However, the prediction accuracy of detecting soil nitrogen content with near infrared sensor is largely influenced by soil water content [15]. Bernard G. Barthès et al. intended to access how near infrared prediction accuracy of carbon and nitrogen content were affected by sample grinding, drying, and replication (use of one to six sub-samples to determine average spectra), the results showed that the predictions were higher with dried, 0.2 mm ground samples [16]. Hernández et al. found that the soil particle size and the soil water content had a great impact on the soil spectral characteristics. When the soil water content was high, it would affect the soil nitrogen estimation accuracy by near infrared sensor [17]. Zeng et al. used the MPA Fourier near infrared sensor to analyze near infrared spectroscopy of purple soil with different water content. The results turned out that under the same organic matter content, the soil absorbance showed a nonlinear upward trend when the soil water content increased, the model had a low prediction accuracy under a high water content as well [18]. Wang et al. used partial least squares to establish the organic prediction model of red soil under different soil water content, suggesting that the correlation between organic matter and first order differential spectroscopy was increased at first and then decreased with the increase of soil water content, which achieved the highest correlation coefficients when the soil water content was 100–150 g/kg [19]. Kuang et al. measured the soil organic carbon and total nitrogen contents by near infrared sensor. In order to explore the influence of soil water content on the detection of organic carbon and total nitrogen, the soil was monitored in real time, natural state, and 24 h of drying. The results showed that the real-time detection had the worst effect on detecting soil nitrogen content, and detection in natural state was the second and 24 h of drying was the best [20]. The results of the above-mentioned researches indicated that the soil water content was one of the key factors affecting the detection results. Although several researchers suggested that the prediction accuracy was low when the soil water content was high, most studies were limited to the impact of soil water on soil nitrogen detection, few researchers propose the specific relationship between the water content and drying time of soil, which is more meaningful for improving the accuracy of real-time detection of soil nitrogen than optimizing the algorithm and spectral data. This study is conducted to explore the correlation between soil drying time and water content, and the effect of soil water content on the detection of soil nitrogen by near infrared sensor. More markedly, it is to demonstrate an optimum soil water content which saves time cost as well as achieves higher prediction accuracy. It is of great value to use near infrared sensor to realize the demands of rapid, real-time, and accurate detection of soil nitrogen content and other nutrients and to develop rapid detection device.

## 2. Materials and Methods

### 2.1. Sample Preparation

The experimental samples were acidic red soil, which was collected from Qingyuan county, Zhejiang province, China. The urea (CO(NH_2_)_2_) was mixed with distilled water to prepare solutions with nitrogen concentrations of 0 g/kg, 0.1 g/kg, 0.15 g/kg, 0.2 g/kg, 0.25 g/kg, 0.3 g/kg, 0.35 g/kg, 0.4 g/kg, and 0.45 g/kg, respectively. Every time 100 g soil samples were mixed and stirred with 15 mL solution. The mixture of soil and solution were pressed into uniform slices of about 100 mm × 100 mm and cut into approximately 10 mm × 10 mm cubes for spectral collection. The segmented soil samples were placed in an oven of 80 degrees Celsius, and 10 samples of each concentration were taken from the first hour to the eighth hour at a time, taking 90 samples for spectral collection at once. The final 90 samples were taken out after drying for 24 h, and the total number of samples was 810. Moreover, another 100 g soil sample was mixed with 15 mL distilled water and pressed into slice as previous, weighing before drying, the weight of soil was measured, and recorded at each drying time. 

### 2.2. Spectrometric Determination

This experiment used a near infrared optical spectrum instrument from Isuzu Optics Corp. Shanghai (China), whose spectral band range, scale rate, and scan times can be set as required. The band range of spectral acquisition is from 900 nm to 1700 nm, which can collect the intensity, reflectivity and absorbance of spectrum. In order to maintain the integrity of the original soil spectrum and the rapidity of the detection process, this experiment collected 400 points of each spectrum and obtained a spectral image at average 3 times per scan. The spectral collection was carried out in a dark environment in removing the impact of the experimental ambient light and fluorescent light on spectral information acquisition. The smooth side of the soil samples was selected for spectral collection, and 810 soil samples were tested.

### 2.3. Data Analysis

Near infrared light is an electromagnetic wave between the mid infrared light and visible light, and its wavelength is 700–1500 nm. The spectral information is derived from O–H, C–H, N–H, and other hydrogen-containing groups’ internal vibration overlap in both multiple frequency and combination frequency, which can reflect the characteristic signal of various organic matter in this spectral region [21,22]. Besides, the spectral information is stable and easy to access. According to Beer-Lambert law, the spectral characteristics will change with the variation of sample component and structure, the spectra derived from different groups varies in the position and intensity of absorption peaks as well [23]. The water of soil has a high absorption coefficient in the near infrared band, whose variation influences the detection of soil nitrogen content by near infrared sensor. In this paper, two different data processing methods were applied to analyze the spectral information [24]. 

#### 2.3.1. Partial Least Squares Method

Partial least squares regression is a commonly used multivariate statistical method, extracting the aggregate variable which interprets the dependent variable most by decomposing and filtering the data in the system [25]. This algorithm takes both independent variables and dependent variables into account, converting the original data into linear and mutually independent new variables by linear transformation, the new variables are also called the principle factor [26]. While extracting the factors that contain more independent variables, the principal factor should also include as much information as possible of the original data. Hence, the flexibility of partial least squares (PLS) makes it possible to establish regression models when the amount of samples is less than the amount of variables, which is one of the least restrictive methods in the multivariate correction [27].

#### 2.3.2. No Information Variable Elimination Method

Uninformative variable elimination (UVE) is first proposed by Centner et al. to improve the algorithm performance of the PLS model. As a modeling method for variable selection, random variables with the same number of spectral variables were added into the modeling spectral matrix to establish a PLS model and obtain the regression coefficient matrix *B*. The method of distinguishing uninformative variables is to add a random noise matrix artificially and set a threshold according to the noise matrix [28,29]. Besides, the data points with no valid information in the spectral matrix were eliminated to reduce the complexity and improve the predictive performance of the model. The algorithm steps are as follows:

(1) Regress the spectral matrix of calibration set *X* (*n* × *m*) and density matrix *Y* (*n* × 1) through PLS, and select the best principle component *f*. The *n* represents the amount of samples in the matrix and *m* represents the amount of wavelength variables in the spectrum. The same as below.
(2) Generate a noise matrix *R* (*n* × *m*) artificially and combine spectral matrix *X* (*n* × *m*) with noise matrix *R* (*n* × *m*) into a new matrix *XR* (*n* × 2*m*). The prior m columns of the matrix are spectral matrix *X* (*n* × *m*), and the latter m columns of the matrix are noise matrix *R* (*n* × *m*).
(3) Regress the matrix *XR* (*n* × 2*m*) and *Y* (*n* × 1) through PLS and obtain the matrix *B* (*n* × 2*m*), consisted of n sets of PLS regression coefficients, where a cross-validation of a sample is removed each time.
(4) Calculate the standard deviations *s* (1 × 2*m*) and the average vector *mean* (1 × 2*m*) of matrix *B* (*n* × 2*m*) by column, and then calculate the ratio of average vector *mean* (1 × 2*m*) of the regression coefficient to its standard deviations *s* (1 × 2*m*). The formula is as follows. *h* (*i*) = *mean* (*i*)/*s* (*i*), *i* = 1, 2…, 2*m*.
(5) Take the maximum absolute value of *h* in the interval of [*m* + 1, 2*m*], *h_max_* = max [abs (*h*)].
(6) Remove the variables corresponding *h* (*i*) < *h_max_* of the spectral matrix *X* (*n* × *m*) in the interval of [1, *m*], the remaining variables are composed of the new matrix *X_UVE_* by the uninformative variable elimination method.

## 3. Results and Discussion

### 3.1. Near Infrared Spectrum Analysis

**Figure 1:**
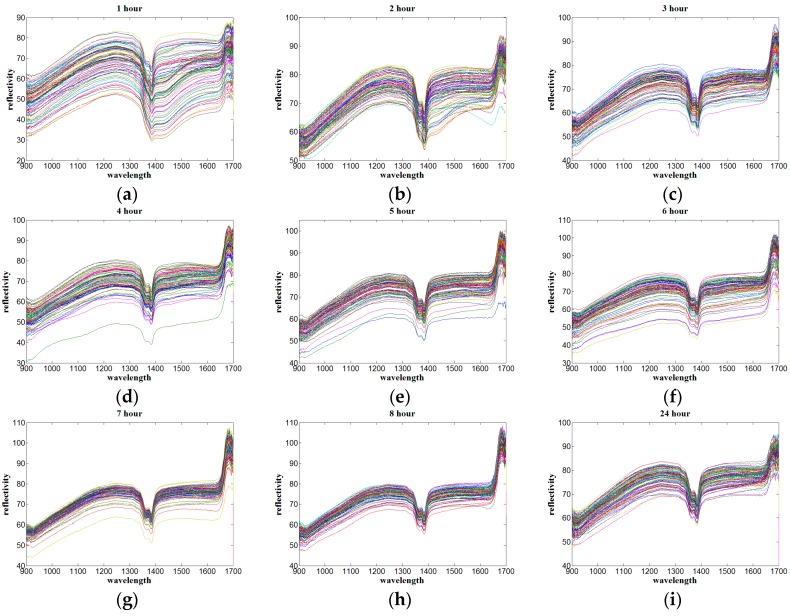
Near infrared reflectivity spectra of soil at different drying time. (**a**–**i**) represent the drying time from 1 h to 8 h and 24 h respectively.

This experiment collected spectral information of soil samples mixed with different nitrogen concentration solutions, where 810 samples were collected at nine kinds of drying time by near infrared sensor. The near infrared reflectivity spectra of the soil collected from 1 h to 8 h and 24 h after drying are shown in Figure 1. 

According to Figure 1, the abscissa of the curve is the wavelength and the ordinate of the curve is the spectral reflectivity. Moreover, each image represents the variation of the reflectance spectra with the wavelength of soil samples at an exact drying time. Firstly, the spectral reflectance from 900 nm to 930 nm, and the band from 1680 nm to 1700 nm vibrates seriously, the reason might be that the near infrared sensor has more spectral information overlap and noise on the edge of the acquisition band. Secondly, the reflectivity of all spectral curves decreases at the band from 1350 nm to 1380 nm, obviously, and there is a maximum absorption peak at around 1380 nm, indicating that, in this band, hydrogen-containing groups of nitrogen such as N–H have a stronger absorption and the nitrogen is more sensitive, which could be considered as the near infrared characteristic band of nitrogen. Thirdly, the reflection peak at around 1680 nm continuously reinforces from 85 to 115 as the drying time increases from 1 h to 8 h, showing that with the decrease of soil water content, the spectral absorption rate is greatly decreased, consequently, and the reflectivity is enhanced. Fourthly, as the increase of drying time, the reflectivity curve becomes more smooth and concentrated in general. The spectral reflectivity intensity is in the range of 30 to 80 after drying for 1 h, and stable in the range of 50 to 110 after drying for 7 h and longer, which suggests that the influence of soil water content on the spectral reflectivity intensity is negatively related. Finally, while the drying time is 3 h, the effect of water on soil nitrogen detection in near infrared band has been reduced to a relatively low level, where the spectral reflectivity curve of soil nitrogen is neither too concentrated nor too dispersed. It demonstrates that at this drying condition, the gradient of the reflectivity curve is more obvious with the increase of soil nitrogen concentration, the detection effect is better as well.

### 3.2. Model and Analysis of Soil Nitrogen Content under Different Drying Time

#### 3.2.1. Partial Least Squares Model

**Figure 2:**
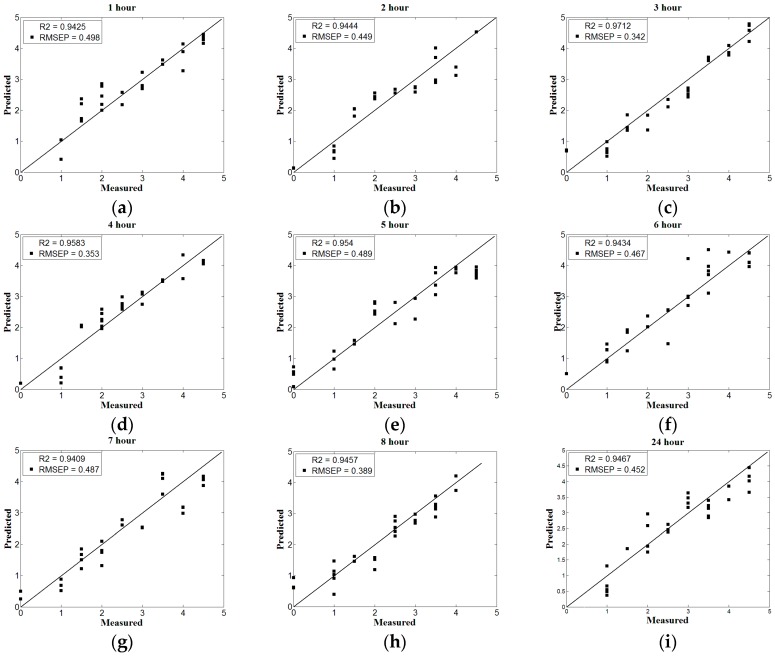
The partial least squares (PLS) model performance under different drying time. (**a**–**i**) represent the drying time from 1 h to 8 h and 24 h respectively.

The prediction model is established with the spectral matrix *X* and the sample physicochemical value *Y*, which reduces the dimension of the computational space to obtain the potential variables and remove the useless variables. In this paper, baseline-corrected and normalized full-wave band spectral data is independent variable *X*, the soil nitrogen content is dependent variable *Y*. Nine PLS nitrogen content prediction models whose drying time is from 1 h to 8 h and 24 h are established and shown in Figure 2. There are 90 samples at each drying time, 60 of which are used for calibration set and 30 of which are for the prediction set. The correlation coefficient R reflects the level of intimacy between the variables, and the root mean square error RMSE reflects the accuracy of the model. The closer the R to 1, and the closer the RMSE to 0, the better the performance and accuracy of the prediction model. Besides, the parameter R1 refers to the correlation coefficient of the calibration set and the parameter R2 refers to the correlation coefficient of the prediction set. The formula for the correlation coefficient R is as follows:

(1) $$R=\frac{\sum_{i=1}^{n}\left(x_{i}-\bar{x}\right)\left(y_{i}-\bar{y}\right)}{\sqrt{\sum_{i=1}^{n}{\left(x_{i}-\bar{x}\right)}^{2}\cdot \sum_{i=1}^{n}{\left(y_{i}-\bar{y}\right)}^{2}}}$$

The formula for root mean square error RMSE is as follows:

(2) $$\mathrm{RMSE}=\sqrt{\frac{1}{n}\overset{n}{\underset{i=1}{\sum }}{\left(y_{i}-\bar{y_{i}}\right)}^{2}\;}$$

**Figure 3:**
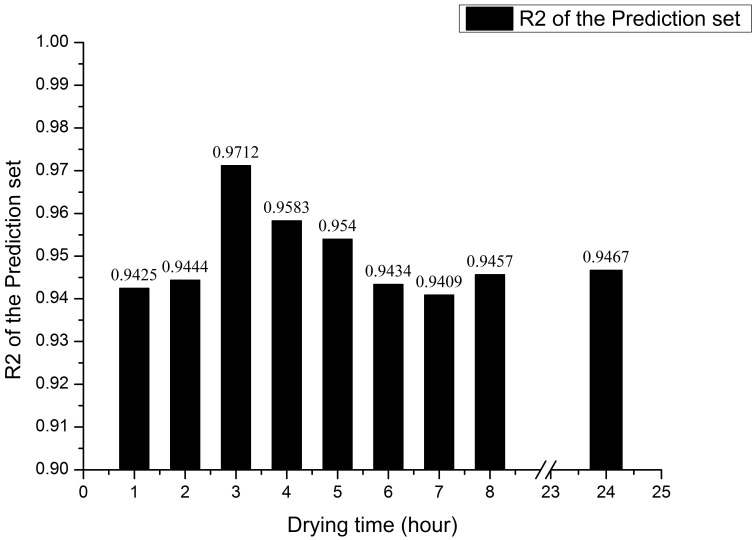
Histogram of PLS model performance under different drying time.

According to the PLS model performance under nine kinds of drying time in Figure 2 and Figure 3, with the increase of drying time, soil water content decreases and the prediction accuracy shows a significant upward trend. From Figure 2c, while the drying time is 3 h, the prediction accuracy is the highest and the correlation coefficient is 0.9712. Then, as the drying time increases, the prediction accuracy of the PLS model decreases slightly and gradually stabilizes after 6 h drying, and the correlation coefficient ranges from 0.94 to 0.95, which is consistent with the prediction accuracy after drying for 24 h. The results suggest that the soil water content changes barely after drying for 6 h and longer, which influences scarcely on the accuracy of prediction models. In conclusion, with the increase of drying time, the accuracy of the PLS model increases first and then decreases, when the drying time is 3 h and the corresponding water content is 1.03%, the soil water content has the lowest negative influence on the detection of soil nitrogen by near infrared sensor.

#### 3.2.2. Uninformative Variable Elimination Method Model

**Figure 4:**
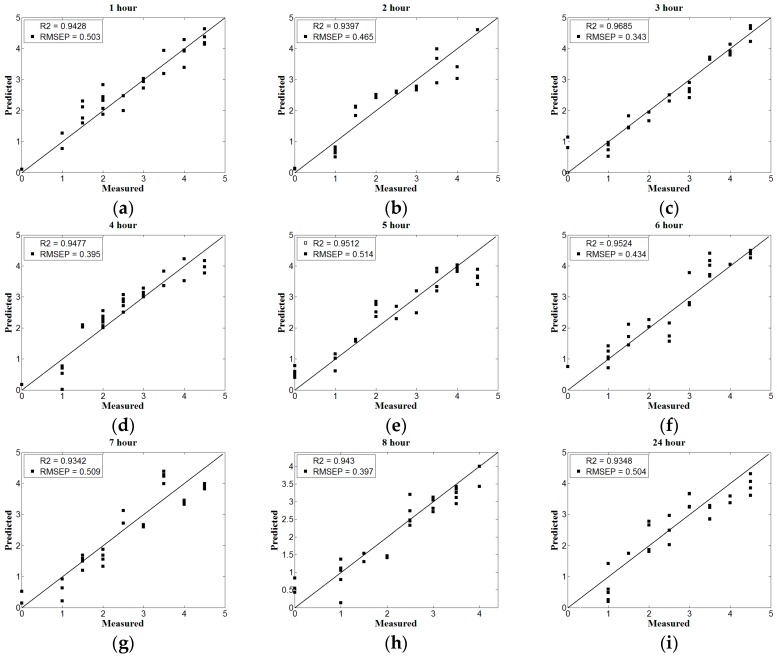
The UVE model performance under different drying time. (**a**–**i**) represent the drying time from 1 h to 8 h and 24 h respectively.

**Figure 5:**
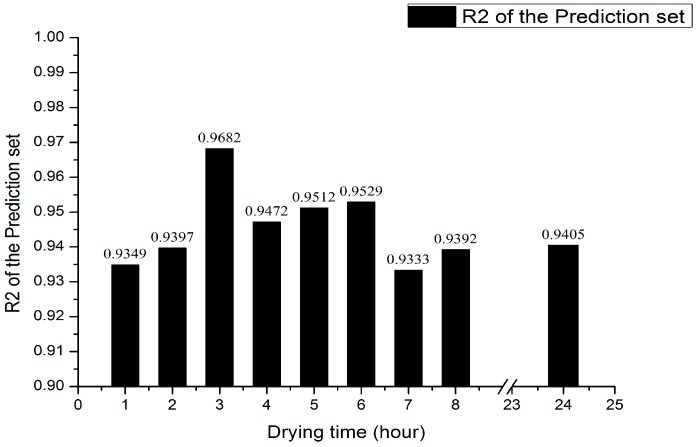
Histogram of uninformative variable elimination (UVE) model performance under different drying time.

The principle component of UVE is determined by the root mean square error (RMSE) of the PLS model, choosing the fraction of the principal variables for further analysis and modeling when the RMSE is minimal and tends to be stable. The nine UVE nitrogen content prediction models from drying for 1 h to 8 h and 24 h are shown in Figure 4 and Figure 5.

According to the UVE model performance under nine kinds of drying time, shown in Figure 4 and Figure 5, as the drying time increases, the prediction accuracy shows a greater upward trend within the 3 h drying, which achieves the highest prediction accuracy when the drying time is 3 h and the corresponding correlation coefficient is 0.9682, according to Figure 4c. Later in the interval of 4 h to 6 h and 7 h to 24 h, the prediction accuracy goes down on the whole, which rises in a small extent within the interval. Overall, the stability of the UVE model is less than that of the PLS model, whose variation trend of the correlation coefficient is basically consistent with that of the PLS model.

#### 3.2.3. Comparison of Two Modeling Methods

**Figure 6:**
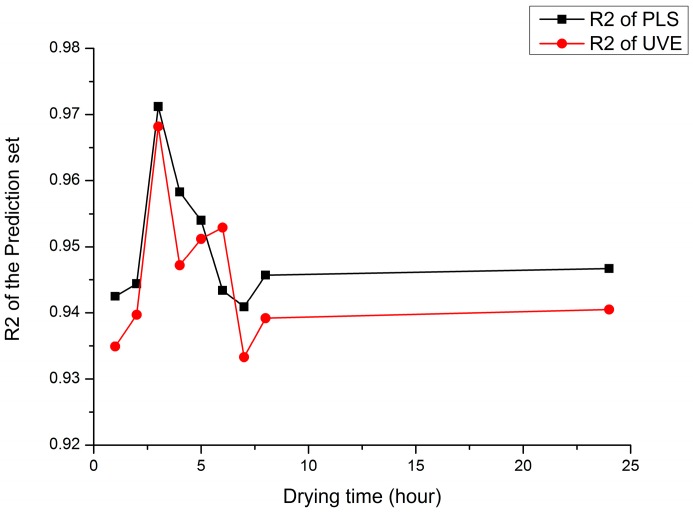
The comparison of modeling performance of PLS and UVE.

This study conducts to explore the effect of dry time and corresponding water content on soil nitrogen content detection by near infrared sensor, using two modeling method including PLS and UVE to model and analyze. The comparison of the PLS model and the UVE model are shown in Figure 6.

According to Figure 6, the prediction models of PLS and UVE both have high correlation coefficients and achieve the highest correlation coefficient of prediction set after drying for 3 h, which is 0.9712 and 0.9682, respectively. When the drying time is 6 h or longer, with the increase of drying time, the correlation coefficient of prediction set tends to be stable. In general, both the prediction accuracy and stability of PLS model are better than that of UVE model, the reason might be that UVE is a derivative algorithm based on the regression coefficient of PLS, which eliminates some of the variables considered to be invalid. However, the immaturity of the UVE algorithm might lead to the elimination of some variables containing effective information, resulting in the lower prediction accuracy of the UVE model when comparing to the PLS model.

### 3.3. Correlative Analysis of Soil Water Content and Modeling Accuracy

The soil water content has a great impact on the detection of nitrogen by near infrared sensor. Based on the current research situation, this study prepares nine kinds of different concentration gradients of nitrogen solutions, fully mixed with the soil. The drying temperature is set to 80 °C and drying time is set from 1 h to 8 h and 24 h, measuring the corresponding soil water content at each drying time. The calculation method of soil water content is given in formula (3).

(3) $$wc=\frac{W_{fresh}-W_{dry}}{W_{dry}}\times 100\%$$

Among them, wc is the soil water content, $W_{fresh}$ is the current weight of soil, and $W_{dry}$ is the final dry weight of soil.

**Figure 7:**
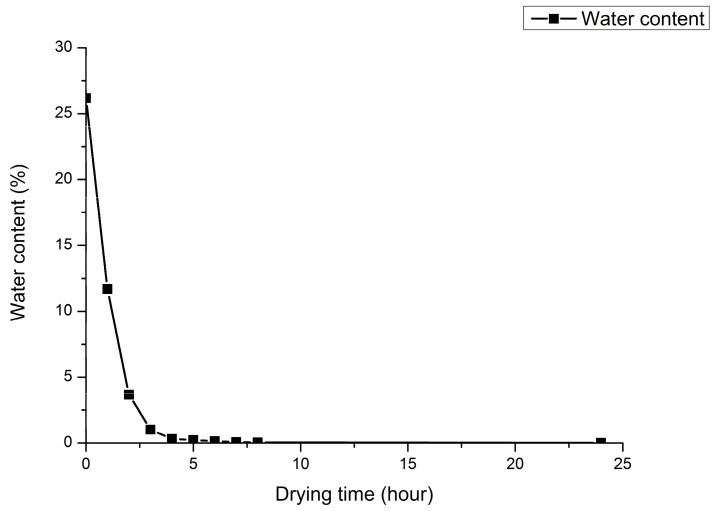
The relationship between soil water content and drying time.

The relationship between soil water content and drying time is shown in Figure 7. The original soil water content (100 g soil sample mixed with 15 mL solution) is 26.17% and drying for 1 h is 11.67%, within 3 h, the soil water content decreases obviously as the drying time increases. After drying for 3 h, the soil water content is 1.03%. When the drying time is from 3 h to 6 h, the soil water content changes little but is still in a gentle downward range. In the interval of 6 h to 8 h, the change of soil water content can be neglected, which has little influence on the detection of soil nitrogen, and there is almost no difference with the effect of 24 h drying, basically it can be determined that the soil is in a completely dry state after drying for 6 h or longer.

The detailed information of drying time, water content, and the modeling effect at different drying times is shown in Table 1. First, when the drying time is 3 h, not only the highest prediction accuracy is achieved, but also the difference of correlation coefficients between the calibration set and the prediction set is very small, while the difference of correlation coefficients between the calibration set and the prediction set at 1 h is relatively big, which indicates that a high water content might influence the over-fitting of PLS and UVE. Second, with the increase of drying time, the decreasing trend of soil water content is rapid within 3 h, and then gradually slows down and changes little after drying for 6 h, which is basically consistent with the variation trend of prediction accuracy. When the drying time is 3 h and the corresponding water content is 1.03%, both PLS and UVE have achieved the optimum modeling effect. Third, some researches proved that the model had a low prediction accuracy under a high water content, which is in accordance with the prediction accuracy at 1 h, however, both correlation coefficients of the PLS and the UVE model at 7 h is a bit lower than that of 1 h, when comparing with the spectrum curve in Figure 1g, indicating that spectral data processing before modeling is necessary for removing abnormal curve as well as improving modeling effect.

## 4. Conclusions

This study was conducted to investigate the optimum soil water content on the detection of soil nitrogen using near infrared sensor. Partial least squares and uninformative variable elimination were used to mathematically model and analyze the correlation between spectral data and soil nitrogen. The main conclusions are as follows:

First, different elements have different reflection characteristics in the near infrared spectral region, the spectral reflectivity curve at 1350 to 1380 nm has a significant decline trend at each drying time, demonstrating that hydrogen-containing groups of nitrogen, such as N–H, have a stronger absorption and nitrogen is more sensitive in this band, which can be considered as the near-infrared characteristic band of nitrogen.

Second, both PLS and UVE performs well in predicting nitrogen content and achieving the highest correlation coefficient of prediction set after drying for 3 h, while the prediction accuracy and stability of the PLS model is better than that of the UVE model, which is more suitable in soil nitrogen prediction combined with near infrared sensor. 

Third, there is a close correlation between soil water content and modeling accuracy. With the increase of drying time, the decreasing trend of soil water content is rapid within 3 h and then gradually slows down and changes little after drying for 6 h, which is basically consistent with the variation trend of modeling accuracy. Moreover, the soil was not treated with sieving and grinding in this study, which meets the prediction requirements under different soil water content, as well as provides a new idea for real-time detection of soil nitrogen in field condition by near infrared sensor.

## Figures and Tables

**Table 1:** Comparison of soil water content and modeling accuracy at different drying time.

Drying Time	Water Content	Methods	R1 of the Calibration Set	R2 of the Prediction Set	Calibration Set RMSEC	Prediction Set RMSEP
1 h	11.67%	PLS	0.9649	0.9425	0.3646	0.498
		UVE	0.9803	0.9349	0.2743	0.528
2 h	3.67%	PLS	0.9554	0.9444	0.4107	0.449
		UVE	0.958	0.9397	0.3991	0.465
3 h	1.03%	PLS	0.9721	0.9712	0.3235	0.342
		UVE	0.9656	0.9682	0.3584	0.344
4 h	0.32%	PLS	0.971	0.9583	0.3491	0.353
		UVE	0.96	0.9472	0.4088	0.397
5 h	0.23%	PLS	0.9338	0.954	0.4694	0.489
		UVE	0.9266	0.9512	0.4936	0.514
6 h	0.14%	PLS	0.9939	0.9434	0.1534	0.467
		UVE	0.9635	0.9529	0.3725	0.433
7 h	0.07%	PLS	0.9301	0.9409	0.5084	0.487
		UVE	0.9431	0.9333	0.4604	0.508
8 h	0.04%	PLS	0.9706	0.9457	0.3551	0.389
		UVE	0.9652	0.9392	0.3835	0.42
24 h	0.01%	PLS	0.9436	0.9467	0.467	0.452
		UVE	0.946	0.9405	0.4571	0.48
